# Topics of the Month

**Published:** 1892-11

**Authors:** 


### TOPICS OF THE MONTH.

THE *United States Investor*, a business weekly, published in Boston, offers prizes amounting to $1,000 for essays, of not more than one column each, respecting American cities and towns. The judges to award these prizes are Hon. Henry Cabot Lodge, of Massachusetts; Hon. Charles F. Crisp, of Georgia; and the Hon. Julius C. Burrows, of Michigan. The prizes will be subdivided as follows: For the best essay respecting any American city or town, $500; for the second best essay on the same subject, $300; and for the third best, $200. Here is an opportunity for public-spirited citizens to write something about Buffalo that will give it distinction. It would seem very easy for Buffalo to take the first prize in this contest when the subject is so large and attractive. The essays that are intended for competition should be marked as such and forwarded to either of the offices of the *Investor*, namely, 19 Pearl street, Boston; 335 Broadway, New York; or 241 Chestnut street, Philadelphia.

---

THE *Electrical Review*, which has been heretofore devoted to the consideration of mechanical and applied electricity, has established a medical department, for the purpose of considering the therapeutical uses of electricity. Dr. J. Mount Bleyer is appointed to conduct this part of the *Review*, and a prospectus has been issued to the medical profession calling attention to this fact. The *Review*, in establishing a medical branch, follows in the wake of one or two other commercial journals, and we can take no exception to this action from the business side of the question; but we desire to enter our protest, most emphatically, against any countenancing this method of journalism by the profession. One medical editor of a lay journal now holds himself up as the very apostle of medical ethics, and yet we submit that in thus appearing once a week before the public as an editor of a medical column in a lay journal, that goes into business quarters far more than into professional circles, he is violating the very foundation principle of all professional ethics. The New York State Medical Association, which is the self-constituted guardian of medical ethics as formulated and expounded by the Judicial Council of the American Medical Association, should appoint a committee to investigate the medical editors of lay journals.

---

*La Revista Médico-Quirúrgica Americana* is the title of a new monthly medical publication printed in New York, and edited by Drs. Samuel E. Milliken and Pedro J. Salicrup. A long list of collaborators is announced, embracing the names of some of the prominent members of the profession. There are some strange juxtapositions of opinion with reference to medical ethics amongst the staff of this new journal, which is the authorized organ in the Spanish language of the Pan-American Medical Congress. For instance, here we see the names of Dr. Henry G. Piffard, of New York, and Dr. N. S. Davis, of Chicago; of Dr. Albert L. Gihon, Medical Director of the United States Navy, and Dr. John A. Wyeth, of New York; of Dr. Joseph Price, of Philadelphia, and Dr. Lewis A. Sayre, of New York; of Dr. David Webster, of New York, and Dr. Henry O. Marcy, of Boston. These names represent the very antipodes of medical ethics. Verily, medical journalism, as well as politics, makes strange bedfellows.

---

MEDICAL spelling and pronunciation is one of the themes of the hour. The tendency at present is to simplify and Americanize as much as possible. Hence, in the journalistic world we see diphthongs dropped whenever they can be with propriety, and pronunciation is made to follow the simplest, which are always the best, rules. The chemical section of the American Association for the Advancement of Science, at its annual meeting in 1887, appointed a committee to make revision in its spelling and pronunciation. Under the chairmanship of Dr. T. H. Norton, of Cincinnati, this committee pursued its work during four years, consulting the leading medical philologists as well as the American chemists in general, and finally reported to the Association at its meeting in 1891. In an article published in the *Popular Science Monthly* for September, 1892, by Frederik A. Fernald, is an interesting statement of the work of the committee, and a discussion of the subject of the pronunciation and spelling of chemical and geographical words. It will look somewhat strange to the eye to substitute f for ph in sulfur and all its derivatives, but we have no doubt it will soon come to be the accepted method of spelling. It is quite necessary for an American to speak and write American as far as possible ; hence, the growing tendency is to Anglicize words of Latin and Greek origin whenever practicable to do so. This is another improvement that advanced thinkers and writers are struggling to establish.

---

The *Sanitarian* for October, 1892, has fairly overreached itself in its criticism of the Government quarantine order issued under the sanction and with the approval of the President. This venerable sanitary magazine says that the President's signature was secured to the most disgraceful edict against practical sanitation that has emanated from any official entrusted with the duty of protecting public health during the last thirty years. Then, if it were possible to do so, it adds insult to injury by publishing the order with a mourning border. It must be that the editor of the *Sanitarian* has lost his head, for it does not seem probable that such a disgraceful exhibition of partisan hatred could have escaped the pen of any right-minded journalist.

---

THE *Indiana Medical Journal* has recently put itself into a new dress, and infused into its columns an activity and method that is worthy of commendation. This journal is easily regarded as one of the foremost medical magazines in the West. We congratulate the editors on their progressive and enterprising spirit.

---

THE *Annals of Ophthalmology and Otology* announces its removal from Kansas City to St. Louis, Mo. This quarterly journal of practical ophthalmology, otology, laryngology, and rhinology, will soon enter its second year of publication, and has become the leading organ of the specialties that it represents. It is handsomely printed and ably edited.

---

THE Committee on Organization of the Pan-American Medical Congress will issue the preliminary announcement of the Congress within a few days. This announcement will show that the organization has been perfected in almost every colony and country of the Western Hemisphere. The local medical societies in each of the constituent countries are made auxiliary to the Congress, which will be held in Washington, D. C., September 5, 6, 7, and 8, 1893. Dr. Charles A. L. Reed, of Cincinnati, Secretary-General of the Congress and Chairman of the Committee on Organization, announces that, after extended correspondence between himself and Dr. Maragliano, of Genoa, General Secretary of the Eleventh International Medical Congress, the date of the Rome meeting has been finally and definitely set for September 24th, of next year. This gives an interval of sixteen days between the Washington and Rome meetings, during which time an easy trip can be made from the former to the latter city. It is possible that a steamer may be chartered direct from New York to Rome.

